# Differential Effects of Interruptions and Distractions on Working Memory Processes in an ERP Study

**DOI:** 10.3389/fnhum.2020.00084

**Published:** 2020-03-16

**Authors:** Bianca Zickerick, Sven Thönes, S. Oliver Kobald, Edmund Wascher, Daniel Schneider, Kristina Küper

**Affiliations:** ^1^Leibniz Research Centre for Working Environment and Human Factors, TU Dortmund, Dortmund, Germany; ^2^Experimental Psychology, Department of Psychology, Johannes Gutenberg University Mainz, Mainz, Germany; ^3^Bundeswehr Institute for Preventive Medicine, Koblenz, Germany

**Keywords:** external interference, interruption, distraction, working memory, attention, EEG

## Abstract

Interruptions (interfering stimuli to respond to) and distractions (interfering stimuli to be ignored) have been shown to negatively impact performance, particularly in tasks requiring working memory (WM). This study investigated how these two types of external interference affect task performance and attentional and WM processes as indexed by specific event-related potentials (ERPs) of the EEG. A Continuous Number Task (CNT) was applied, in which participants had to either decide whether the current number (condition without WM load) or the sum of the current and the preceding number (condition with WM load) was odd or even while responding to interlaced single letters (interruptions) or ignoring them (distractions). Contrary to previous research, we did not find external interference to affect performance under WM load. Unexpectedly, our results rather show that performance was significantly improved in trials after distractions compared to before. This effect was reflected particularly in a significantly increased P3 mean amplitude indicating enhanced attentional reallocation to task-relevant stimuli. Interestingly, this P3 effect appeared independent of WM load and also following interruptions. This underpins the account of P3 amplitudes being modulated by the interval between two task-relevant stimuli rather than by overall task-difficulty. Moreover, a pronounced fronto-central and posterior slow wave following interference suggest more control resources to maintain task-relevant stimuli in WM independent of the preceding interfering stimulus. Our results thus suggest that the type and foreknowledge of external interference may modulate the amount of interference and may also facilitate resource preparation under WM load.

## Introduction

In everyday life and modern working environments, it has become essential to effectively deal with distractions and interruptions. According to Clapp et al. (2010), interruptions are defined as interfering stimuli that require attention, such as a secondary task (e.g., phone calls), whereas distractions describe interfering irrelevant stimuli that capture attention but have to be ignored (e.g., background noise). Attentional and working memory (WM) processes are crucial to efficiently handle these two types of external interference. WM comprises executive control functions, such as the ability to inhibit irrelevant information, as well as the ability to successfully rehearse and maintain information that is relevant for a task (Baddeley, 2012). Attentional processes facilitate these functions as they support the selection of task-relevant information and the recovery from attentional capture by task-irrelevant information (Gazzaley et al., 2005; Cowan, 2008; Gazzaley and Nobre, 2012; Sawaki et al., 2012). This can be referred to as a result of top–down, or goal-directed, attention in contrast to bottom–up, or stimulus-driven, attention (Desimone and Duncan, 1995). Both of these cognitive processes have been shown to be strained by distractions and interruptions leading to impaired performance in a primary task. Particularly in tasks requiring WM, external interference negatively affects the maintenance of task-relevant information (Baddeley, 1986; Logie et al., 1990; Sakai, 2003; Yoon et al., 2006; Gazzaley et al., 2007; Berry et al., 2009; Clapp et al., 2010; Mishra et al., 2013; Schneider et al., 2017; Barth and Schneider, 2018) and hampers the suppression of information that is irrelevant for a task (Vogel et al., 2005; Gazzaley et al., 2008; Zanto and Gazzaley, 2009; Clapp et al., 2010). Previous research has shown that interruptions have an even more detrimental effect on WM performance than distractions (Clapp et al., 2010; Solesio-Jofre et al., 2011, 2012; Clapp and Gazzaley, 2012; Mishra et al., 2013). However, the cognitive processes underlying these distinct performance deficits are as of yet not well-understood. In the present study, we thus aimed to identify how attentional and WM processes are affected differently by interruptions and distractions.

Regarding distractions, previous research has frequently reported that the presence of distracting information captures attention and impairs behavioral performance in WM tasks. In particular, distractions can lead to higher error rates as shown by West (1999), who instructed participants to ignore distractions (e.g., a star-like figure) while memorizing the location of a target in one of four boxes. Zanto and Gazzaley (2009) used a delayed-recognition paradigm, in which participants were instructed to remember the color or motion of an object while ignoring irrelevant stimuli. Behavioral analyses revealed an increase in response times due to the distracting stimuli. Furthermore, the negative effects of distractions increased with increasing memory load in the WM task. Such distraction-related performance decline has been investigated further by examining event-related potentials (ERPs) of the electroencephalogram (EEG) that are associated with the processing of the distracting stimuli. Markers of attentional allocation, such as the early visual ERPs P1 and N1 (Hillyard et al., 1998; Zanto and Gazzaley, 2009) point to limited capacities to inhibit irrelevant information in early stages of visual processing due to the allocation of a substantial amount of attention to the distracting information (Vogel et al., 2005; Gazzaley et al., 2008; Zanto and Gazzaley, 2009; Clapp et al., 2010).

With respect to interruptions, only few EEG studies have been conducted, but a large number of behavioral studies has also demonstrated the disruptive effects of interruptions on performance in a primary task (Gillie and Broadbent, 1989; Edwards and Gronlund, 1998; Altmann and Trafton, 2002; Speier et al., 2003; Bailey and Konstan, 2006). Kreifeldt and McCarthy (1981), for example, reported that interruptions (arithmetic tasks) caused longer response times in calculator-based tests due to more time needed to reorient to the primary task. Moreover, in a verbal WM task, Sakai et al. (2002a) showed that interruptions in the form of a secondary arithmetic task impaired the ability to maintain task-relevant information in WM and therefore lead to increased error rates in memory trials in contrast to a non-interfering arithmetic task. Additionally, also MEG studies have shown that particularly interruptions lead to disruptive effects regarding encoding (García-Pacios et al., 2013), maintenance (Solesio-Jofre et al., 2011) and the retrieval of task-relevant information of the primary task (Solesio-Jofre et al., 2012).

A recent study by Bae and Luck (2018) has offered insights into the mechanisms underlying these performance deficits. The authors demonstrated that interruptions distort task-relevant information of the primary task even in simple visual WM tasks. They assumed that stored information within the focus of attention was eliminated due to interruptions. Another explanation for the disruptive effect of interruptions is that they require a reallocation of cognitive resources and demand processes enabling the reactivation of information relevant to the primary task (Sakai et al., 2002b; Clapp et al., 2010). This has been shown in a study using EEG and functional magnetic resonance imaging (fMRI) by Clapp et al. (2010), which is one of the few studies simultaneously addressing the impact of both types of external interference on task performance. They applied a delayed-recognition task, in which interfering face and scene stimuli were presented during the memory-maintenance period. These interfering stimuli either had to be ignored (distractions) or responded to (interruptions). As the authors were primarily interested in the processing of both types of external interference, ERP analyses were conducted to identify markers of attentional allocation during the processing of interruptions and distractions. In line with previous research on the effects of distractions, their results indicated that both types of external interference affected WM performance during the early stages of visual processing as indexed by modulations of markers of attentional allocation, such as the early visual P1. In particular, their findings indicate that an increased allocation of attention toward distractions and interruptions impairs WM performance. Interestingly, the behavioral data suggested that interruptions had an even more detrimental effect on WM performance than distractions (see also Solesio-Jofre et al., 2011, 2012; Mishra et al., 2013). Clapp et al. (2010) therefore assumed different cognitive mechanisms underlying the processing of distractions and interruptions. This was supported by fMRI evidence from their study indicating that task-relevant information was maintained during the processing of distractions but had to be reactivated after an interruption. This dissociation points toward the relevance of investigating the processing of stimuli following an interference.

Therefore, the current study did not focus on the processing of interruptions and distractions *per se* but investigated the effects of interruptions and distractions on the processing of subsequent stimuli of the primary task. More precisely, our aim was to examine differential effects on task performance and attentional and WM processes after interruptions and distractions compared to before an interference, as indexed by specific event-related EEG potentials. To this end, we examined a Continuous Number Task (CNT). In this task, participants had to make an odd-or-even decision on single numbers presented continuously in a random sequence. In some experimental blocks, they additionally had to ignore intermittent single letters (distractions) or respond to such letters (interruptions) in a subset of trials. Participants performed task blocks with WM load (rehearsing a number during interference) as well as without WM load (no rehearsal required). As a control condition, in some trials of distinct experimental blocks, instead of an interrupting or distracting stimulus, the fixation cross was prolonged resulting in equal trial durations in all experimental conditions. The present CNT thus allows for a continuous investigation of task performance contingent on different types of interfering stimuli and different levels of WM load.

As interferences necessitate a reallocation of cognitive resources, we focused on the posterior P3 component as a measure of top-down mediated attentional allocation (Polich, 2007) and processing capacity (Kok, 2001). Previous research has shown that the P3 is modulated by task-relevance (Duncan-Johnson and Donchin, 1977; Gazzaley et al., 2008; Getzmann et al., 2018). Consequently, interrupting stimuli that require attention should have an effect on the posterior P3 (P3b) in trials following an interruption affecting top–down attentional processes. Moreover, they should affect cognitive control for subsequent trials as interrupting stimuli require a reallocation of cognitive resources in order to retrieve task-relevant information of the primary task (Sakai et al., 2002b; Clapp et al., 2010). As a marker of cognitive control, we thus investigated the fronto-central N2 component (Kopp et al., 1996; Bartholow et al., 2005; Folstein and Van Petten, 2008). Furthermore, we examined the fronto-central slow wave as an index of WM maintenance that is modulated by interfering stimuli (Ruchkin et al., 1995; Vogel et al., 2005; Fukuda et al., 2015). In particular, the fronto-central slow wave is thought to reflect processes of cognitive control that are necessary to maintain task-relevant information (Mecklinger and Pfeifer, 1996; Bosch et al., 2001). If interruptions indeed disrupt the maintenance of task-relevant information, they should thus diminish this ERP index after an interruption occurs. As the main task in the condition with WM load does not only require the maintenance of task-relevant information but also further cognitive operations, we additionally investigated the posterior slow wave which has been associated with cognitive operations following target identification (Johnson and Donchin, 1985; Ruchkin et al., 1988) and therefore should be also affected in trials following interference.

In general, we hypothesized that both types of external interference negatively impact task performance in subsequent trials in contrast to trials without a preceding interference. We assumed that this impact would be even more detrimental for interruptions than for distractions since interruptions as a secondary task (1) demand more attentional resources, (2) disrupt the ability to maintain task-relevant information in WM and (3) require processes to reactivate task-relevant information afterward. Accordingly, we expected the effects of interruptions to be more pronounced in the condition with WM load as compared to the control condition without WM load. Modulations of P3 and N2 amplitudes in trials after interruptions should reflect this decline in performance on the electrophysiological level revealing impaired attentional and cognitive control processes. This should be especially pronounced in the condition with WM load. In the condition with WM load, we further expected the posterior and fronto-central slow wave to be substantially reduced, especially after an interruption, reflecting impaired WM processes. We thus assumed that both, attentional and WM processes, were impaired after an external interference with greater declines following interruptions.

## Materials and Methods

### Participants

Twenty-two healthy adults took part in the experiment. According to the criterion for far outliers proposed by Tukey (1977), data from five participants were excluded from the analyses (mean individual accuracy scores ± 3 SDs from sample mean in two or more conditions). Data from another participant had to be discarded due to an excessive amount of missing responses (97% in one condition). This fairly high exclusion rate can be justified in consideration of the rather complex and difficult WM task used in the experiment.

The remaining sixteen adults (*M* = 24 years, *SD* = 2.96 years; range = 19 – 28 years; 8 females) had normal or corrected-to-normal vision, were right-handed according to a handedness questionnaire (adapted from Oldfield, 1971) and reported to be free of medication. All participants gave their written informed consent and received course credit or a payment of 10€ per hour. The study was conducted according to the Code of Ethics of the World Medical Association (Declaration of Helsinki) and was approved by the local Ethics Committee of the Leibniz Research Centre for Working Environment and Human Factors, Dortmund, Germany.

### Apparatus and Stimuli

Participants were tested individually in an electrically shielded, dimly lit EEG chamber. All stimuli were presented at a viewing distance of 145 cm on a 22-inch CRT monitor with a refresh rate of 100 Hz and a resolution of 1024 × 786 pixels. The experimental task was programmed using Lazarus IDE (Free Pascal) and stimulus presentation was controlled by a ViSaGe MKII Stimulus Generator (Cambridge Research Systems, Rochester, United Kingdom). All experimental stimuli were displayed against a gray background with a luminance of 10 cd and subtended a visual angle of 2° height. Numbers from 1 to 6 (standard stimuli) were presented in white Arial font with a luminance of 80 cd. Additionally, the letters “U” and “V” and the letters “M” and “N” (interfering stimuli) were presented in the same font in two distinct subsets of experimental blocks. The letters “U”/“V” and “M”/“N” were chosen as potential target stimuli due to their visual similarity to each other and because they were easily distinguishable from the numbers 1 to 6 and the fixation cross. The assignment of numbers and letters to the different experimental conditions was counterbalanced between participants. Each number and each letter was presented pseudo-randomized with equal frequency throughout the experiment. Responses were given via two force keys that were attached to the right and left armrest of the participant’s chair.

### Task and Procedure

In the CNT, participants had to either decide whether the current number (condition without WM load) or the sum of the current and the preceding number (condition with WM load) was odd or even. Additionally, in a subset of experimental blocks, 25% of the standard stimuli were replaced by interfering stimuli that required the participants to either respond to single letters (interruptions), ignore single letters (distractions) or wait for a longer delay period before the presentation of the next standard stimulus (prolonged fixation cross).

Prior to the beginning of each block, instruction slides were presented to the participants to inform them about which task they had to perform in the upcoming block. In order to get acquainted with the CNT, all participants first completed a block of 150 standard trials without interfering stimuli and without WM load. A given trial consisted of a fixation cross that was followed by either a standard stimulus (standard trial) or an interfering stimulus (interference trial with interruptions, distractions or prolonged fixation crosses). The following six blocks were presented randomly under the condition that two consecutive blocks could not feature the same interfering stimuli in order to reduce potential training effects.

Overall, the experimental session comprised two blocks with interruptions, two blocks with distractions and two blocks with prolonged fixation crosses. The participants’ task changed randomly between blocks: in one half of the blocks, participants had to decide whether the current number was odd or even (condition without WM load), whereas in the other half of the blocks they had to decide whether the sum of the current and the preceding number was odd or even (condition with WM load) by responding with the left or right force key (assignment of response keys was counterbalanced between participants). Crucially, in the condition with WM load, the number preceding an interfering stimulus had to be memorized before adding it to the number following the interfering stimulus.

**FIGURE 1:**
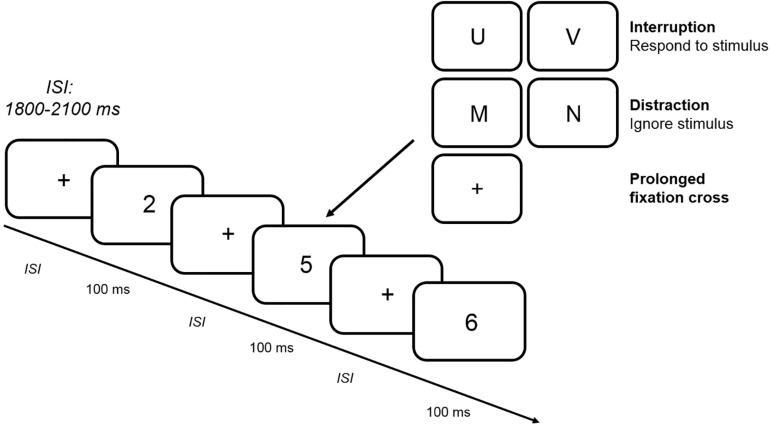
Trial procedure. The figure depicts an example of a random sequence of standard stimuli presented in the CNT. In a subset of blocks (two interruption blocks with and without WM load, two distraction blocks with and without WM load and two prolonged fixation cross blocks with and without WM load), 25% of all standard stimuli were replaced by interfering stimuli (interruption vs. distraction vs. prolonged fixation cross). While participants had to ignore the letter in a distraction trial and wait for a longer delay period in a prolonged fixation cross trial, a response was required in an interruption trial. *ISI*: interstimulus interval.

Figure 1 illustrates the experimental procedure. Each standard trial began with the jittered presentation of a fixation cross for between 1800 and 2100 ms, which was followed by the presentation of a number from 1 to 6 for 100 ms. After the participant’s response, the next standard trial began. In a block with interruptions, the number was replaced by a letter that was presented for 100 ms after the presentation of a fixation cross in 25% of all trials. Participants had to respond to the letter by pressing the left or right force key (assignment of response keys was counterbalanced between participants). In contrast to interruption trials, participants had to ignore the letter that was presented for 100 ms in a distraction trial. In a prolonged fixation cross trial, the number was replaced by a fixation cross for additional 100 ms. All trials containing an interfering stimulus (interruption, distraction or prolonged fixation cross) were followed by a standard trial. In total, each block comprised 320 trials with 80 interference trials resulting in an experimental session lasting between 3 and 3 1/2 h including the preparation for the EEG recording. Between each of the six blocks, short breaks of 2–5 min had to be taken in order to prevent fatigue during the experiment.

### Behavioral Data Recording and Analyses

Button presses with a force of at least 150 cN were registered as responses. Response errors comprised fast guesses (responses faster than 100 ms after target onset), missing responses (no responses or responses slower than 1500 ms after target onset) as well as incorrect button presses. The first five trials of each block were excluded from the analyses in order to reduce the impact of adaptation effects. For each participant, the mean accuracy and the mean response times of correct responses (RT) were computed separately for the three different interference trials as well as for the two WM load conditions and separately for all trials before and the trial directly after an interference. Thus, the experimental design comprised three within-subject factors: *WM load* (with WM load vs. without WM load), *Interference Type* (interruption vs. distraction vs. prolonged fixation cross) and *Trial Type* (all trials before (x−n) vs. one directly after (x+1) an interference). Accuracy and RT served as dependent variables and were further analyzed in separate repeated-measures analyses of variance (rm-ANOVAs) with Greenhouse–Geisser correction for the degrees of freedom in cases where sphericity could not be assumed (as indicated by Greenhouse-Geisser ε). Partial eta squared ($\eta_{\text{p}}^{2})$ is reported as an estimator of effect size. *Post hoc* analyses were conducted by the false discovery rate (FDR) procedure by Cramer et al. (2016) in order to correct for cumulation of Type 1 error within the ANOVAs. In these instances, critical *p*-values were adjusted (*p*_crit_). Additionally, *post hoc* comparisons were performed using the FDR procedure for multiple comparisons with adjusted *p*-values (denoted as *p*_adj_).

### EEG Data Recording and Analyses

EEG activity was recorded from 64 Ag/AgCl electrodes (Easycap; Brain Products, Gilching, Germany). Electrodes were arranged according to the extended 10/20 System (Pivik et al., 1993). A 2 × 32 Channel NeurOne Tesla AC-amplifier (Bittium Biosignals Ltd., Kuopio, Finland) was used for data recording. EEG data were low-pass-filtered at 250 Hz and recorded at a sampling rate of 1000 Hz. Impedances of all electrodes were kept below 10 kΩ. The midline electrode AFz served as ground electrode, whereas FCz served as the reference electrode.

Offline analyses of the EEG data were conducted using the EEGLAB (Delorme and Makeig, 2004) toolbox for MATLAB (Mathworks, Natick, MA, United States). EEG data were band-pass filtered with a high-pass filter of 1 and a 40 Hz low-pass-filter. After rejecting bad channels with kurtosis exceeding 10 SD (*M* = 2.31 channels, *SD* = 2.06) using a channel rejection tool imbedded in EEGLAB, EEG data were re-referenced to average reference. Segments with a length of 3300 ms (−700 to 2600 ms) were extracted, down-sampled to 250 Hz and submitted to an independent component analysis (ICA). The ADJUST procedure (an automatic algorithm for EEG artifact removal) was used to automatically detect ICs containing artifacts, such as generic data discontinuities, eye movements or eye blinks. Identified ICs were then removed from the dataset. Additionally, the EEGLAB toolbox plug-in DIPFIT identified cortical dipoles for all ICs and removed ICs from the dataset when the unexplained variance of the dipole exceeded 50%. Subsequently, the IC structure was projected onto the 1000 Hz data. In preparation for the ERP analyses, the EEG data were bandpass-filtered at 0.1 Hz – 40 Hz and segmented again into epochs from −200 to 2600 ms. Only trials with correct responses before (x−n) and directly after an interference (x+1) were considered for the ERP analyses. In a trial sequence in which a standard trial directly followed an interfering stimulus (x+1) and concurrently appeared before another interference (x−n), the trial was considered as an x+1 trial as participants were not aware of the following trial at this point in time. Trials with artifacts were rejected by an automatic artifact rejection implemented in EEGLAB (threshold limit: 1000 μV, probability threshold: 5 SD, Max.% of trials rejected per iteration: 5%). On average, 705 trials (*SD* = 126.6) were rejected from the ERP analyses. As for the behavioral data, the first five trials of each block were excluded from further analyses. ERPs were averaged separately for *Trial Type* (x−n vs. x+1), *WM load* (with WM load vs. without WM load) and *Interference Type* (interruption vs. distraction vs. prolonged fixation cross). This resulted in a grand average ERP, meaning the average waveforms for each condition averaged across participants.

The following stimulus-locked ERP components were investigated: The N2 was analyzed by first determining the peak latency between 200 – 350 ms at electrode Fz in the grand average. Then, the mean amplitude was computed in a time window around the most negative peak (condition with WM load: 317 ms; ± 50 ms; condition without WM: 311 ms; ±50 ms). For the posterior P3, peak latencies were measured within a 300 ms time window (350 – 650 ms), and the mean amplitude was computed in the resulting time windows around the most positive peak (condition with WM load: 516 ms; ±100 ms; condition without WM load: 494 ms; ±100 ms). The fronto-central slow wave was measured as the mean amplitude between 800 – 1200 ms at Fz. For the posterior slow wave, we determined the same time window as for the frontal slow wave and analyzed the mean amplitude at electrode Pz. Moreover, we also analyzed sensory ERP components to test for an imbalance of early sensory (bottom–up; see Desimone and Duncan, 1995) processing between the experimental conditions. The posterior P1 and N1 were measured at the mean of electrodes PO7 and PO8 as peak latency (P1: 50 – 100 ms; N1: 100 – 200 ms) and as mean amplitude within a 40 ms time window. The P1 was centered on the most positive peak (condition with WM load: 95 ms; ±20 ms; condition without WM load: 96 ms; ±20 ms) and the N1 was computed in the time window around the most negative peak in the grand average (condition with WM load: 144 ms; ±20 ms; condition without WM load: 143 ms; ±20 ms). For each component rm-ANOVAs were performed including the factors *Interference Type*, *Trial Type*, and *WM load*. Mean amplitudes and peak latencies served as dependent variables in this regard. Within these ANOVAs, the false discovery rate procedure was used for correcting for cumulation of Type 1 error (as indicated by adjusted critical p-values *p*_crit_). Partial eta squared ($\eta_{\text{p}}^{2})$ is reported as an estimator of effect size. Interactions were decomposed by *post hoc* analyses of ANOVA. In follow-up analyses, *p*-values were FDR-corrected for multiple comparisons as indicated by *p*_adj_.

## Results

### Behavioral Data

**FIGURE 2:**
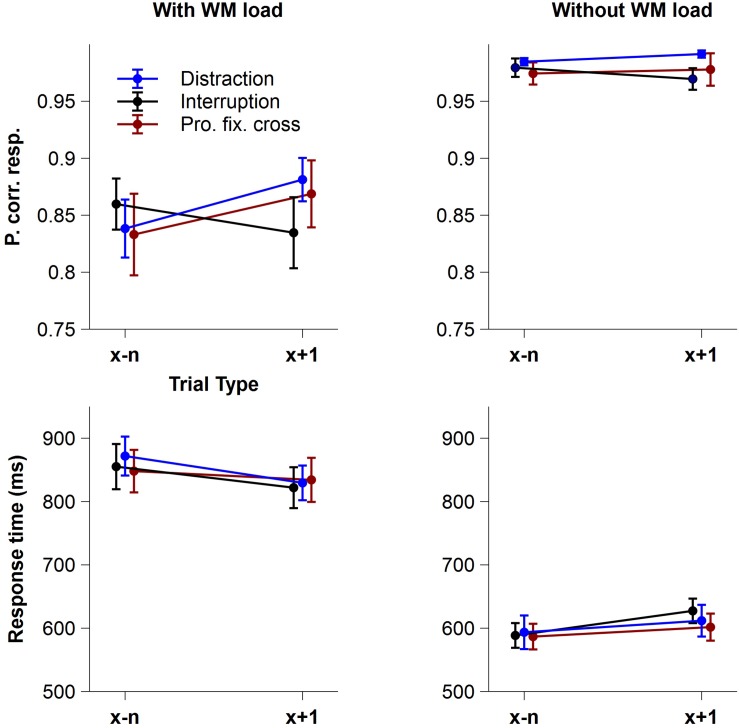
Behavioral data. Mean proportion of correct responses (accuracy) and mean response times of correct responses (RT) as a function of *Trial Type* and *Interference Type*, separately for the conditions with and without WM load. Blue lines display trials before and after a distraction, black lines trials before and after an interruption and red lines trials before and after a prolonged fixation cross. Error bars indicate ± 1 standard error of the mean (SEM).

Figure 2 shows the mean proportion of correct responses (accuracy) and the mean response times of correct responses (RT) as a function of *Trial Type* and *Interference Type*, separately for the condition with and without WM load.

Regarding the proportion of correct responses, the rm-ANOVA revealed a significant main effect of *WM load*, indicating that participants responded more accurately in the condition without WM load (*M* = 0.98, *SD* = 0.03) compared to the condition with WM load (*M* = 0.85, *SD* = 0.11), *F*(1,15) = 41.77, *p* < 0.001, *p*_crit_ = 0.05, $\eta_{\text{p}}^{2}$ = 0.74. Neither the factor *Trial Type*, *F*(1,15) = 3.50, *p* = 0.081, *p*_crit_ = 0.021, $\eta_{\text{p}}^{2}$ = 0.19, nor *Interference Type, F*(1,15) = 0.40, ε = 0.695, *p* = 0.604, *p*_crit_ = 0.014, $\eta_{\text{p}}^{2}$ = 0.03, showed significant main effects, but the interaction of *Interference Type x Trial Type* did, *F*(2,30) = 7.45, *p* = 0.002, *p*_crit_ = 0.043, $\eta_{\text{p}}^{2}$ = 0.33, indicating different effects of distractions, interruptions and prolonged fixation crosses on accuracy in trials following an interference. Importantly, this interaction was further modulated by *WM load* resulting in a significant 3-way *Trial Type x Interference Type x WM load* interaction, *F*(2,30) = 4.34, *p* = 0.022, *p*_crit_ = 0.036, $\eta_{\text{p}}^{2}$ = 0.22. *Post hoc* analyses showed significant *Trial Type* differences in the condition with WM load, *F*(2,30) = 6.68, *p* = 0.004, *p*_crit_ = 0.05, $\eta_{\text{p}}^{2}$ = 0.31, but not in the condition without WM load, *F*(2,30) = 2.74, *p* = 0.081, *p*_crit_ = 0.05, $\eta_{\text{p}}^{2}$ = 0.15. When WM load was required, task accuracy significantly improved in trials following distractions, *F*(1,15) = 6.81, *p*_adj_ = 0.030, $\eta_{\text{p}}^{2}$ = 0.31, and prolonged fixation crosses, *F*(1,15) = 9.15, *p*_adj_ = 0.027, $\eta_{\text{p}}^{2}$ = 0.38, compared to trials before these interferences. Unexpectedly, we did not find an effect following interruptions, *F*(1,15) = 2.57, *p*_adj_ = 0.130, $\eta_{\text{p}}^{2}$ = 0.15. None of the remaining interactions for accuracy reached statistical significance [*Trial Type x WM load*: *F*(1,15) = 3.62, *p* = 0.076, *p*_crit_ = 0.029, $\eta_{\text{p}}^{2}$ = 0.19; *Interference Type x WM load*: *F*(2,30) = 0.01, *p* = 0.994, *p*_crit_ = 0.007, $\eta_{\text{p}}^{2\,}$ < 0.01].

With respect to response times, participants were significantly slower in the condition with WM load (*M* = 843.43 ms, *SD* = 127.82 ms) than without WM load (*M* = 601.61 ms, *SD* = 87.59 ms), *F*(1,15) = 167.06, *p* < 0.001, *p*_crit_ = 0.05, $\eta_{\text{p}}^{2}$ = 0.92. *Interference Type, F*(2,30) = 0.24, *p* = 0.786, *p*_crit_ = 0.007, $\eta_{\text{p}}^{2}$ = 0.02, and *Trial Type, F*(1,15) = 0.17, *p* = 0.690, *p*_crit_ = 0.021, $\eta_{\text{p}}^{2}$ = 0.01, showed no significant effects. However, there was a significant *Trial Type x WM load* interaction, *F*(1,15) = 27.26, *p* < 0.001, *p*_crit_ = 0.043, $\eta_{\text{p}}^{2}$ = 0.65, which was further modulated by the type of interference, as indicated by the significant three-way *Trial Type x Interference Type x WM load* interaction, *F*(2,30) = 4.71, *p* = 0.017, *p*_crit_ = 0.036, $\eta_{\text{p}}^{2}$ = 0.24. *Post hoc* analyses revealed only marginally significant differences in the condition without WM load. Here, the interaction of *Trial Type* and *Interference Type*, *F*(2,30) = 3.43, *p* = 0.046, *p*_crit_ = 0.033, $\eta_{\text{p}}^{2}$ = 0.19, indicated slower responses particularly in trials after an interruption compared to before one when no WM load was required. None of the remaining interactions for RT reached statistical significance (all *p* > 0.251).

### EEG Data

#### Sensory ERP Components

None of the investigated factors *Trial Type*, *F*(1,15) = 1.02, *p* = 0.328, *p*_crit_ = 0.036, $\eta_{\text{p}}^{2}$ = 0.06, *Interference Type, F*(2,30) = 2.99, *p* = 0.066, *p*_crit_ = 0.005, $\eta_{\text{p}}^{2}$ = 0.17, or *WM load, F*(1,15) = 0.03, *p* = 0.869, *p*_crit_ = 0.007, $\eta_{\text{p}}^{2}$ < 0.01, varied for P1 amplitude. Also, the respective interactions did not reach statistical significance (all *p* > 0.242). Moreover, P1 latency did not reveal significant main effects of *Trial Type*, *F*(1,15) = 0.02, *p* = 0.881, *p*_crit_ = 0.007, $\eta_{\text{p}}^{2}$ < 0.01, or *Interference Type*, *F*(2,30) = 0.68, *p* = 0.515, *p*_crit_ = 0.029, $\eta_{\text{p}}^{2}$ = 0.04, but the main effect of *WM load* showed a trend toward later latencies in the condition with WM load compared to the condition without WM load, *F*(1,15) = 4.39, *p* = 0.053, *p*_crit_ = 0.05, $\eta_{\text{p}}^{2}$ = 0.23. For P1 latency, no other two-way (all *p* > 0.496) or three-way interaction [*Trial Type x Interference Type x WM load*: *F*(2,30) = 3.24, *p* = 0.075, *p*_crit_ = 0.043, $\eta_{\text{p}}^{2}$ = 0.18] was statistically significant.

Yet, the sensory component N1 showed significantly smaller mean amplitudes in trials following an interference, *F*(1,15) = 5.19, *p* = 0.038, *p*_crit_ = 0.05, $\eta_{\text{p}}^{2}$ = 0.26, as well as significantly later latencies in trials after an interference, *F*(1,15) = 5.99, *p* = 0.027, *p*_crit_ = 0.05, $\eta_{\text{p}}^{2}$ = 0.29. Neither *Interference Type* nor *WM load* did vary for N1 mean amplitude [*Interference Type: F*(2,30) = 0.28, *p* = 0.654, *p*_crit_ = 0.014, $\eta_{\text{p}}^{2}$ = 0.02; *WM load*: *F*(1,15) = 0.67, *p* = 0.427, *p*_crit_ = 0.021, $\eta_{\text{p}}^{2}$ = 0.04] or N1 latency [*Interference Type*: *F*(2,30) = 2.72, *p* = 0.082, *p*_crit_ = 0.043, $\eta_{\text{p}}^{2}$ = 0.15; *WM load*: *F*(1,15) = 0.02, *p* = 0.901, *p*_crit_ = 0.014, $\eta_{\text{p}}^{2}$ < 0.01]. None of the remaining interactions for N1 mean amplitude and peak latency was statistically significant (all *p* > 0.170).

#### N2 Mean Amplitude

**FIGURE 3:**
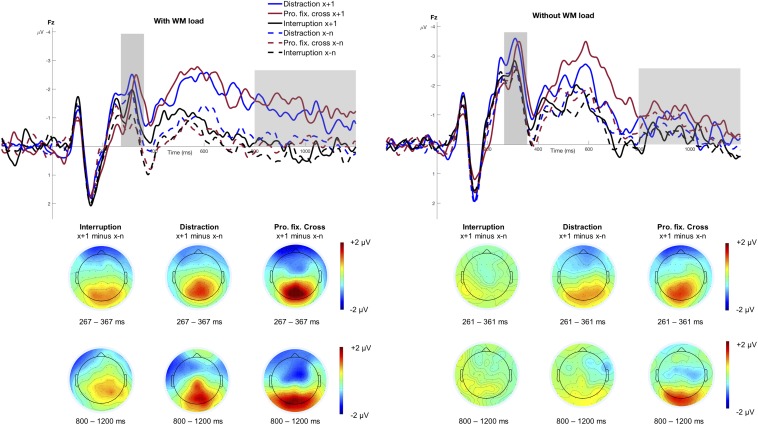
Grand averages at electrode Fz. The figure displays the stimulus-locked grand averages at electrode Fz as a function of *Trial Type* and *Interference Type* separately for the conditions with and without WM load. The dashed lines illustrate all trials before an interference (x−n) and the solid lines refer to trials that directly followed an interference (x+1). ERPs associated with distractions are depicted as blue, interruptions as black and the prolonged fixation cross as red lines. Positive deflections are displayed downward. The gray areas highlight the analyzed time windows of the N2 mean amplitude (condition with WM load: 267 – 367 ms; condition without WM load: 261 – 361 ms) and the fronto-central slow wave (800 – 1200 ms). The scalp topographies display the difference between the trial after an interference (x+1) and all trials before an interference (x−n), separately for the three different interference types.

Figure 3 shows the stimulus-locked grand averages at electrode Fz as a function of *Trial Type* and *Interference Type*, separately for the condition with and without WM load. The figure highlights the N2 mean amplitude that was significantly larger for trials following an interference (*M* = −2.00 μV, *SD* = 2.23 μV) than those preceding it (*M* = −1.36 μV, *SD* = 1.94 μV), *F*(1,15) = 21.03, *p* < 0.001, *p*_crit_ = 0.043, $\eta_{\text{p}}^{2}$ = 0.58. Moreover, the N2 was significantly larger when no WM load was involved (*M* = −2.23 μV, *SD* = 2.18 μV) compared to the condition with WM load (*M* = −1.14 μV, *SD* = 1.89 μV), *F*(1,15) = 24.36, *p* < 0.001, *p*_crit_ = 0.05, $\eta_{\text{p}}^{2}$ = 0.62. The main effect of *Interference Type* reached only marginally significance, *F*(2,30) = 3.36, *p* = 0.048, *p*_crit_ = 0.036, $\eta_{\text{p}}^{2}$ = 0.18, indicating that the N2 was increased in blocks involving distractions (*M* = −1.96 μV, *SD* = 2.01 μV) and prolonged fixation crosses (*M* = −1.64 μV, *SD* = 2.38 μV) as compared to interruptions (*M* = −1.45 μV, *SD* = 1.90 μV). None of the remaining interactions regarding the N2 mean amplitude reached statistical significance (all *p* > 0.339).

#### N2 Peak Latency

N2 peak latency did not vary with *Trial Type*, *F*(1,15) = 4.08, *p* = 0.062, *p*_crit_ = 0.036, $\eta_{\text{p}}^{2}$ = 0.21, *Interference Type, F*(2,30) = 0.85, *p* = 0.436, *p*_crit_ = 0.014, $\eta_{\text{p}}^{2}$ = 0.05, or *WM load, F*(1,15) = 0.61, *p* = 0.449, *p*_crit_ = 0.007, $\eta_{\text{p}}^{2}$ = 0.04. However, it did vary between the three different interference types in trials following an interference, *F*(2,30) = 5.81, *p* = 0.007, *p*_crit_ = 0.05, $\eta_{\text{p}}^{2}$ = 0.28. This interaction was further modulated by *WM load*, *F*(2,30) = 4.17, *p* = 0.025, *p*_crit_ = 0.043, $\eta_{\text{p}}^{2}$ = 0.22. *Post hoc* analyses revealed that peak latencies occurred significantly later in the condition with WM load, *F*(2,30) = 6.56, *p* = 0.004, *p*_crit_ = 0.05, $\eta_{\text{p}}^{2}$ = 0.30, but not in the condition without WM load, *F*(2,30) = 1.19, *p*_adj_ = 0.317, *p*_crit_ = 0.043, $\eta_{\text{p}}^{2}$ = 0.07. In particular, N2 peak latencies were significantly delayed in trials after a prolonged fixations cross compared to trials before when WM load was required, *F*(1,15) = 9.68, *p*_adj_ = 0.021, $\eta_{\text{p}}^{2}$ = 0.39. This result pattern might indicate a disrupted work flow due to the prolonged fixation cross. However, N2 latencies did not differ significantly in trials following interruptions, *F*(1,15) = 0.30, *p*_adj_ = 0.594, $\eta_{\text{p}}^{2}$ = 0.02, and distractions, *F*(1,15) = 0.48, *p*_adj_ = 0.594, $\eta_{\text{p}}^{2}$ = 0.03. None of the remaining interactions was statistically significant (all *p* > 0.130).

Overall, it should be noted that the N2 might rather reflect a rising flank of the P3 when taking into account the respective topography in Figure 3. Therefore, the focus of our discussion will lie primarily on the P3 component which is reported below.

#### P3 Mean Amplitude

**FIGURE 4:**
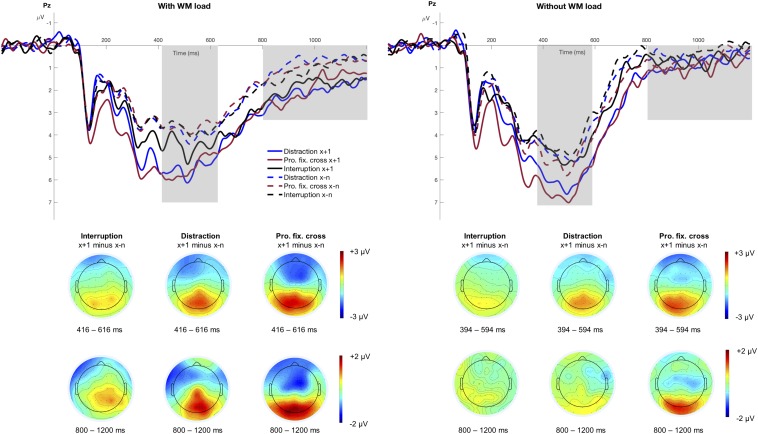
Grand averages at electrode Pz. The figure illustrates the stimulus-locked grand averages at electrode Pz as a function of *Trial Type* and *Interference Type* separately for the conditions with and without WM load. Dashed lines illustrate all trials before an interference (x−n), solid lines indicate the trial that directly followed an interference (x+1). ERPs associated with distractions are depicted as blue, interruptions as black and the prolonged fixation cross as red lines. Positive deflections are displayed downward. The gray area highlights the analyzed time window of the P3 component (condition with WM load: 416 – 616 ms; condition without WM load: 394 – 594 ms) and the posterior slow wave (800 – 1200 ms). The scalp topographies are based on the difference between the trial after an interference (x+1) minus all trials before an interference (x−n), respectively for the three different interference types.

Separate stimulus-locked grand averages at electrode Pz for the different factors of *WM load*, *Trial Type* and *Interference Type*, are depicted in Figure 4. The figure suggests a pronounced P3 after an interference, especially in blocks with distractions and prolonged fixation crosses. The significant main effect of *Trial Type*, *F*(1,15) = 65.53, *p* < 0.001, *p*_crit_ = 0.05, $\eta_{\text{p}}^{2}$ = 0.81, indicated increased P3 mean amplitudes in trials following an interference (*M* = 5.47 μV, *SD* = 2.97 μV) relative to trials preceding it (*M* = 4.24 μV, *SD* = 2.58 μV). Furthermore, the P3 amplitude significantly differed between interruptions, distractions, and prolonged fixation crosses as indicated by the main effect of *Interference Type*, *F*(2,30) = 6.98, *p* = 0.003, *p*_crit_ = 0.043, $\eta_{\text{p}}^{2}$ = 0.32. This effect was especially pronounced in trials after an interference as indicated by a significant *Trial Type x Interference Type* interaction, *F*(2,30) = 4.04, *p* = 0.028, *p*_crit_ = 0.036, $\eta_{\text{p}}^{2}$ = 0.21. Interestingly, *post hoc* analyses revealed that the P3 mean amplitude was significantly increased after all three interference types, distractions, *F*(1,15) = 24.36, *p*_adj_ < 0.001, $\eta_{\text{p}}^{2}$ = 0.62, prolonged fixation crosses, *F*(1,15) = 35.60, *p*_adj_ < 0.001, $\eta_{\text{p}}^{2}$ = 0.70, and also in trials following interruptions, *F*(1,15) = 8.76, *p*_adj_ = 0.010, $\eta_{\text{p}}^{2}$ = 0.37. Since we did not find a main effect neither of *WM load*, *F*(1,15) = 4.23, *p* = 0.058, *p*_crit_ = 0.029 $\eta_{\text{p}}^{2}$ = 0.22, nor any corresponding interaction (all *p* > 0.109), the increase in P3 amplitude seems to be unaffected by modulations of WM load in our experimental design, although the respective topographies point to stronger differences in the condition with WM load (see Figure 4).

#### P3 Peak Latency

Neither for *Trial Type*, *F*(1,15) = 3.27, *p* = 0.090, *p*_crit_ = 0.043, $\eta_{\text{p}}^{2}$ = 0.18, nor *Interference Type, F*(2,30) = 1.72, *p* = 0.195, *p*_crit_ = 0.029, $\eta_{\text{p}}^{2}$ = 0.10, nor *WM load*, *F*(1,15) = 1.11, *p* = 0.308, *p*_crit_ = 0.014, $\eta_{\text{p}}^{2}$ = 0.07, P3 peak latency varied with the task. The interaction *Trial Type x WM load* indicated only marginal differences, *F*(1,15) = 4.51, *p* = 0.051, *p*_crit_ = 0.05, $\eta_{\text{p}}^{2}$ = 0.23, suggesting earlier latencies in trials following an interference when WM load was required. None of the remaining interactions reached statistical significance (all *p* > 0.168).

#### Posterior Slow Wave

The posterior slow wave is illustrated in Figure 4 between 800 and 1200 ms and was significantly larger in trials after an interference (*M* = 1.34 μV, *SD* = 2.16 μV) compared to before (*M* = 0.69 μV, *SD* = 1.65 μV), *F*(1,15) = 15.49, *p* = 0.001, *p*_crit_ = 0.05, $\eta_{\text{p}}^{2\,}$ = 0.51, and also significantly more pronounced in the condition with WM load (*M* = 1.41 μV, *SD* = 2.11 μV) compared to the condition without WM load (*M* = 0.62 μV, *SD* = 1.68 μV), *F*(1,15) = 9.39, *p* = 0.008, *p*_crit_ = 0.043, $\eta_{\text{p}}^{2\,}$ = 0.39. Furthermore, the interaction *Trial Type x WM load*, *F*(1,15) = 4.81, *p* = 0.045, *p*_crit_ = 0.036, $\eta_{\text{p}}^{2\,}$ = 0.24, reached marginal significance indicating an increased posterior slow wave in trials after an interference when WM load was demanded, *F*(1,15) = 15.37, *p*_adj_ = 0.002, $\eta_{\text{p}}^{2\,}$ = 0.51, but not in the condition without WM load, *F*(1,15) = 2.42, *p*_adj_ = 0.141, $\eta_{\text{p}}^{2\,}$ = 0.14. This effect underlines our assumption of the posterior slow wave reflecting a cognitive operation that was required only in the condition with WM load.

As there were no differences for *Interference Type*, *F*(2,30) = 0.45, *p* = 0.642, *p*_crit_ = 0.021, $\eta_{\text{p}}^{2}$ = 0.03, nor any corresponding interaction with that factor (all *p* > 0.636), the posterior slow wave does not seem to be affected by the three different interference types.

#### Fronto-Central Slow Wave

Figure 3 illustrates the fronto-central slow wave between 800 and 1200 ms. The rm-ANOVA revealed a significant main effect of *Interference Type*, *F*(2,30) = 7.56, ε = 0.737, *p* = 0.006, *p*_crit_ = 0.05, $\eta_{\text{p}}^{2}$ = 0.34, indicating larger slow waves in a block with distractions (*M* = −0.51 μV, *SD* = 2.17 μV) and prolonged fixation crosses (*M* = −0.73 μV, *SD* = 1.75 μV) compared to interruptions (*M* = 0.03 μV, *SD* = 1.66 μV). The analysis showed no main effect of *WM load*, *F*(1,15) = 0.02, *p* = 0.882, *p*_crit_ = 0.007, $\eta_{\text{p}}^{2\,}$ < 0.01, or *Trial Type*, *F*(1,15) = 4.14, *p* = 0.060, *p*_crit_ = 0.036, $\eta_{\text{p}}^{2\,}$ = 0.22, but revealed a significant *Trial Type x WM load* interaction, *F*(1,15) = 7.75, *p* = 0.014, *p*_crit_ = 0.043, $\eta_{\text{p}}^{2}$ = 0.34. *Post hoc* analyses indicated a significantly larger slow wave in trials following an interference in the condition with WM load, *F*(1,15) = 7.07, *p*_adj_ = 0.036, $\eta_{\text{p}}^{2}$ = 0.32, but not in the condition without WM load, *F*(1,15) = 0.86, *p*_adj_ = 0.367, $\eta_{\text{p}}^{2}$ = 0.05, as this ERP component is related to WM processes. No other significant interactions regarding the fronto-central slow wave were found (all *p* > 0.106).

## Discussion

The main aim of the present study was to investigate how interruptions and distractions affect attentional and WM processes. To this end, we examined behavioral performance and ERPs on trials before an interference in contrast to directly after an interference in a CNT. This task allowed for investigating the influence of randomly presented interfering stimuli on task performance in conditions with and without WM load.

We hypothesized that both types of external interference would negatively affect task performance and cognitive processes particularly when WM load was involved. However, our results point to rather opposite results revealing positive effects of distractions in the condition with WM load as indexed by increased task accuracy. This effect was also reflected in modulations of the posterior P3, an ERP correlate of attentional allocation suggesting a pronounced enhancement of mean amplitude in response to stimuli following a distraction. Additionally, also interruptions did not reveal a negative effect when WM load was required, neither on task performance nor at the electrophysiological level.

To clarify the reasons for these results, we first need to take a look at the behavioral level. As hypothesized, participants performed significantly worse in the condition with WM load indicating that performing an arithmetic task demands more cognitive resources than merely responding to a single number. Consequently, we expected to see greater impairments following interruptions and distractions when WM load was involved as previous studies have shown (Fockert et al., 2001; Clapp et al., 2010; Mishra et al., 2013). Interestingly, responses in the primary task tended to be slower after interruptions compared to before, exclusively in the condition without WM load. This may indicate that an interrupted workflow delays the reallocation of attention to the primary task even in a simple task, supposedly without further involvement of WM processes (see also Bae and Luck, 2018). In contrast, distractions did not show an effect in the condition without WM load. This result pattern might be related to the fact that the primary task as well as the distracting stimuli were too simple and required no attentional or WM processes (Craik, 2014). In keeping with this, we observed a ceiling effect with respect to task accuracy when no WM load was demanded.

However, what we did find was an effect of distractions on task performance in the condition with WM load. According to Lavie’s load theory (Lavie et al., 2004), whenever WM load is demanded and therefore less cognitive resources are available, this effect leads to increased distractor interference. Surprisingly, our results point in the opposite direction revealing a benefit from distracting information under WM load as reflected in increased task accuracy in trials following distractions. Further exploration of the literature offers indeed evidence for either no effect or even a beneficial effect of distractions (Yi et al., 2004; Kim et al., 2005; Scheiter et al., 2014). For instance, Park et al. (2007) reported distractions to facilitate target selection under concurrent WM load in a selective attention task. They argue that the extent to which a person successfully selects and inhibits information depends on the amount of shared common resources of the relevant and irrelevant information in a task. This is in line with the multiple capacity framework proposed by Wickens (2002), claiming that the similarity of tasks within the dimensions *stages of processing, codes, and modalities*, determines the amount of task interference. Accordingly, presenting distracting stimuli that are different from the stimuli in the primary task would result only in little interference (Rae and Perfect, 2014). This multiple resource theory might also offer a possible explanation for the positive effects of distractions in our study. Here, we presented highly dissimilar standard and distracting stimuli that were easily distinguishable. In accordance with that, we found a similar result pattern following distractions and prolonged fixation crosses indicating that distractions might have served more as a pause releasing further cognitive resources.

This assumption of increased available resources following distractions is affirmed on the electrophysiological level. In general, we found enhanced electrophysiological markers of cognitive control in trials following an interference as indexed by larger amplitudes of the fronto-central N2 (Folstein and Van Petten, 2008), although attenuated N1 amplitudes and later N1 latencies point to hampered selective attention toward the stimulus after an interference (Mangun, 1995; Hillyard and Anllo-Vento, 1998). These findings may indicate that participants paid less attention to the “x+1” trial at its appearance, but were still capable of enabling control resources as participants were aware of the task-relevant stimuli following an interference and could prepare for the upcoming trial.

Moreover, we found an increased fronto-central slow wave in trials after an interfering stimulus. As expected, this effect was found only in the condition with WM load since the magnitude of frontal slow waves is scaled according to WM load (Ruchkin et al., 1995; Bosch et al., 2001; Vogel and Machizawa, 2004) and the maintenance of information was not required in the condition without WM load. In accordance with studies suggesting that mid-frontal slow waves reflect WM maintenance as well as cognitive control processes (Mecklinger and Pfeifer, 1996; Bosch et al., 2001), the increased slow wave may indicate improved WM maintenance and rehearsal activity. This may be due to more available control resources, which further resulted in increased task performance following distractions and prolonged fixation crosses. In fact, previous studies have already pointed out that task-relevant information was still maintained during the presentation of distractions due to an efficient suppression of the distracting stimuli (Sakai et al., 2002a; Sakai, 2003; Clapp et al., 2010) and active rehearsal of task-relevant information. This was reflected in prefrontal cortex (PFC) activity, which has been shown to play an essential role in maintaining task-relevant information (O’Reilly, 2002; Sakai et al., 2002b; Yoon et al., 2006). However, the increased slow wave may not only reflect the rehearsal of the trial prior to an interference but also of the current “x+1” trial which had to be maintained in order to perform the arithmetic operation in the condition with WM load. This assumption can be confirmed with respect to response times indicating that the slow wave was not only present before but also after initiating the response. Furthermore, the additional cognitive operation was reflected in a posterior slow wave (Ruchkin and Sutton, 1983; Johnson and Donchin, 1985; Garcìa-Larrea and Cézanne-Bert, 1998) that tended to be also pronounced in trials after distractions, interruptions and prolonged fixations crosses. This result suggests that participants rehearsed and maintained the number in WM (as reflected in the fronto-central slow wave) while combining it with the preceding number in order to perform and prepare for the additional cognitive operation (as indexed by the posterior slow wave). As both, the posterior and particularly the fronto-central slow wave were enhanced in trials after an interference independent of the type of interference, this sequence of processing seems to be unaffected by distractions, interruptions and prolonged fixation crosses when WM load was demanded. Instead, the enhancement after an interference suggests increased cognitive resources which may be a consequence of the easily distinguishable stimulus material we have used. Therefore, participants had more time to prepare for the subsequent stimulus while maintaining and rehearsing task-relevant information of the primary task in WM.

This time benefit might have had an even more pronounced effect within the framework of our experimental design than expected, particularly when taking into account the posterior P3 as an index of attentional resources (Isreal et al., 1980; Kok, 2001; Polich, 2007). Regarding the P3, we found significantly increased amplitudes, especially in trials following distractions and prolonged fixation crosses. This result nicely underpins the improvement in task accuracy in the condition with WM load. We assume that participants were in charge of more available attentional resources shifting attention back to the primary task more easily. Surprisingly, the enhancement of the P3 amplitude was observed following all three interference types, independent of WM load. Whereas P3 modulations are typically associated with task-difficulty (Kok, 2001) or stimulus probability (Duncan-Johnson and Donchin, 1977), this observation, in turn, indicates that the time interval between stimuli modulates the P3 amplitude (Gonsalvez and Polich, 2002; Steiner et al., 2013, 2016). In detail, longer intervals between stimuli have been shown to produce greater P3 amplitudes (Steiner et al., 2013) which fits our results of larger P3 amplitudes particularly after distractions and prolonged fixation crosses that did not require a response. Consequently, the time between the two task-relevant stimuli of the primary task increased, as did the amount of available cognitive resources resulting in enhanced task accuracy.

What is quite intriguing to us is that the P3 amplitude did also significantly increase following interruptions compared to trials before interruptions. Recent studies showed that interruptions require more attention and therefore more cognitive resources (Speier et al., 2003; Clapp et al., 2010). This might indicate that interruptions would negatively affect attentional processes of task-relevant stimuli. Considering that we found higher P3 amplitudes assuming even more available resources after interruptions than before might again speak in favor of the timing account (Croft et al., 2003). We assume that participants were able to process the interruption task without difficulty. Regardless of a secondary task, they might have had still enough time to engage attentional resources in preparation for the subsequent task-relevant stimulus.

So far, we can conclude that our results did not reveal negative effects of external interference in a CNT with WM load, neither on attentional nor WM processes. These findings contradict previous research, that showed a decrease in WM performance after both types of external interference, interruptions as well as distractions (Berry et al., 2009; Clapp et al., 2010; Mishra et al., 2013). It is important to note that these studies made use of far more complex stimuli, for example degraded face stimuli that resulted in worse task performance (Yoon et al., 2006; Clapp et al., 2010). Additionally, their interfering stimuli were highly similar to the stimuli of the primary task. Processing similar stimulus material has been shown to disrupt task performance (Kreifeldt and McCarthy, 1981; Gillie and Broadbent, 1989), due to more shared common resources according to the multiple capacity framework (Wickens, 2002). As pointed out above, our stimuli were not only very dissimilar to each other, but assumingly also required fewer attentional demands than for example face stimuli in the study by Clapp et al. (2010). This might be due to the fact that the letters were not as complex as stimulus material in previous studies and hence not disruptive enough to expect a severe impact of interfering stimuli. Given that distracting and interrupting stimuli were presented block-wise and participants were aware of their appearance, this knowledge might have further facilitated interference processing without negatively affecting WM processes (see also Guynn and McDaniel, 2007). These findings support the idea that it is not only WM load that modulates the influence of interference, but rather the type of stimulus material regarding its *similarity*, *attentional demands*, and the *foreknowledge* about its type and appearance. Future studies should thus refer to this account by for example investigating the influence of distractions and interruptions combined in one experimental block. This combination would hamper the distinction between task-relevant and -irrelevant information. Moreover, combining both types of external interference may be even more relevant with regard to applied perspectives, as interruptions and distractions frequently occur simultaneously in everyday life and modern working environments and must be evaluated situationally.

## Conclusion

In this study, we investigated the influence of interruptions and distractions on attentional and WM processes in a CNT with and without WM load. Against our hypotheses, we did not find a negative effect neither of distractions nor interruptions on WM performance and attentional processes. Importantly, our results rather revealed a beneficial effect of distractions and prolonged fixation crosses on task accuracy when WM load was demanded. This effect was reflected particularly in increased P3 amplitudes suggesting that participants were able to successfully reallocate attention to the primary task. Surprisingly, this P3 effect appeared independent of WM load and also following interruptions. This supports the account of P3 amplitudes being predicted by the time interval between two task-relevant stimuli (Croft et al., 2003) rather than by task-difficulty (Kok, 2001) or stimulus probability (Duncan-Johnson and Donchin, 1977). Moreover, this finding suggests that our interfering stimuli may have been easily processed due to the dissimilar stimulus material and a block-wise stimulus presentation. Consequently, participants could build a preset of potential interference and were able to expect and prepare for the subsequent task-relevant stimulus. This, in turn, enabled and facilitated resource preparation leading to more available attentional resources and also better WM maintenance and improved performance of additional cognitive operations as reflected in a pronounced fronto-central and posterior slow wave in trials following an interference. Hence, our results highlight that not merely WM load but the type and knowledge of interfering stimuli might play a crucial role in modulating the impact of interferences. Furthermore, this strengthens the proposal that external interference may also facilitate task performance under given circumstances. Particularly in the context of working environments, these findings are gaining in importance and demonstrate that short breaks or simple distractions might even offer advantages.

## Data Availability Statement

The datasets generated for this study are available on request to the corresponding author.

## Ethics Statement

The studies involving human participants were reviewed and approved by the local Ethics Committee of the Leibniz Research Centre for Working Environment and Human Factors, Dortmund, Germany. The patients/participants provided their written informed consent to participate in this study.

## Author Contributions

KK, EW, ST, and BZ conceived the idea of the study. BZ carried out the experiment. SK and BZ analyzed the data. ST and DS verified the analytical methods. All authors aided in interpreting the results. BZ took the lead in writing the manuscript. All authors contributed to shaping the research, analysis, and manuscript.

## Conflict of Interest

The authors declare that the research was conducted in the absence of any commercial or financial relationships that could be construed as a potential conflict of interest.
